# West Chicago Medical Society

**Published:** 1879-03

**Authors:** 


### Article XI.

The West Chicago Medical Society held its regular meeting at the Washingtonian Home, Feb. 10th, Dr. Bridge in the chair.

Dr. E. W. Lee related a case of fracture of the neck of the femur, in a boy ten years old. After sustaining the injury the lad walked home, a distance of several blocks; indeed, he was able to walk for two days afterwards, not being compelled to keep his bed until the third day after the injury. There was no suspicion of fracture until the third week, at which time Dr. Lee was called in, and discovered an extra-capsular fracture. In response to a question, the doctor stated as his explanation of the case, the presumption that there had been impaction of the shaft which persisted for the time during which the boy used the limb.

In the discussion which followed, Dr. A. H. Tagert mentioned a similar case. A man about 50 years of age was pushed from the platform of a street car, falling in the stony street upon his hip. He arose and walked home, a distance of ten blocks. He was subsequently found to have sustained a fracture of the neck of the femur, the limb being 2 inches shorter than its fellow.

Dr. Bridge exhibited Bartlett's forceps, for application at the superior strait. Several advantages were pointed out, among them the fact that the axis of traction could be easily made parallel to the axis of the superior strait. The instrument was the object of considerable criticism, both favorable and adverse.

Dr. Lee exhibited a splint for fracture of the femur, in which counter-extension was secured by taking advantage of the conical shape of the thigh. No perineal band is needed. The advantages claimed are mobility of the limb, simplicity of dressing, security of the extension. While originally designed for children, it had been used, to the doctor's entire satisfaction, on adults.

